# Socioeconomic and Familial Factors Associated with Gross Motor Skills among US Children Aged 3–5 Years: The 2012 NHANES National Youth Fitness Survey

**DOI:** 10.3390/ijerph17124491

**Published:** 2020-06-22

**Authors:** Soyang Kwon, Meghan O’Neill

**Affiliations:** Ann & Robert H. Lurie Children’s Hospital of Chicago, Chicago, IL 60611, USA; meoneill@luriechildrens.org

**Keywords:** early childhood, preschoolers, locomotor, object control, family income, family structure

## Abstract

The first aim of this study was to examine the prevalence of below average gross motor skills in a representative sample of US children aged 3 to 5 years. The second aim was to identify socioeconomic and familial characteristics that are associated with below average gross motor skills. Secondary analysis was conducted using the datasets from the 2012 National Health and Examination Survey National Youth Fitness Survey (NNYFS). The NNYFS assessed gross motor skills among 329 children aged 3–5 years, using the Test of Gross Motor Development-Second Edition (TGMD-2). Socioeconomic and familial characteristics of interest, such as family income and family structure, were asked in an in-person interview. This study estimated that one in three US children age 3 to 5 years old (33.9%) scored below average for gross motor quotient. In the gross motor subsets, one in four (24.4%) scored below average for locomotion and two in five (39.9%) scored below average for object control. Children living below the poverty threshold were more likely to have a higher gross motor quotient (odds ratio, OR = 2.76; 95% confidence interval, CI = 1.09–7.00). Girls were more likely to have a higher locomotor score (OR = 2.17; 95% CI = 1.10–4.25). Those living with other child(ren) aged ≤5 years were more likely to have a higher locomotor score (OR = 2.36; 95% CI = 1.01–5.54), while those living with child(ren) aged 6–17 years were more likely to have a higher object control score (OR = 1.83; 95% CI = 1.24–2.69). This study revealed risk factors associated with poor gross motor development, furthering our understanding of gross motor development in early childhood.

## 1. Introduction

Gross motor skills involve the large muscles of the body that enable major body movements, such as walking, maintaining balance, coordination, jumping, and reaching [1]. As a subset of gross motor skills, locomotor skills in children include those that require fluid movements of the body as the child moves horizontally or vertically from one place to another, such as walking, running, and jumping. Object control skills are those that demonstrate efficient throwing, striking, and catching movements. Early childhood is a critical period to acquire movement proficiency as children rapidly develop a range of gross motor skills [2]. For example, by age 3, most children can go up and down stairs by alternating their feet, jump in place, and throw overhand. By age 4, most children can catch a bounced ball, jump with a running start, and pedal a tricycle. By age 5, most children can gallop leading with one foot, roll like a log, and propel themselves on a swing. Motor skill competence has been shown to be associated with multiple aspects of health, such as physical activity, cardiovascular fitness, and healthy weight status [3], as well as with language development [4] and academic performance [5].

Understanding the factors that influence motor skill competence is important to nurture young children to have the best chance of developing adequate fundamental motor skills, and to inform movement-based guidelines that can assist parents and clinicians [6]. Studies have investigated the correlates of gross motor skill development in early childhood. Among biological factors, age has been consistently reported to be positively associated with locomotor and object control skills [7,8]. Findings for sex differences in gross motor development have been mixed: some studies [7,9,10,11], but not all [12,13,14], reported higher object control skills among boys, while some studies [10], but not all [9,13,14,15], reported higher locomotor skills among girls. Obesity was reported to be associated with poorer bodyweight-related motor skills, such as hopping and jumping, among 4 year old children [16]. Birthweight has also been investigated, but no significant association with gross motor development was found [17,18]. Among behavioral factors, lower screen time and higher physical activity levels have been shown to be associated with higher gross motor competence among preschool-aged children [19,20,21,22,23]. As differences in socioeconomic status often translate into inequities in child development across many counties/societies [24], socioeconomic status has also been suggested to be positively associated with gross motor skills [11,25], though the data on this have been somewhat conflicting [26] or weak. Some studies [12,27] have accounted for the impact of socioeconomic status on gross motor outcomes in statistical modeling, but the impact was not clearly presented. Other family-level factors have also been investigated. For example, living with other children of a similar age or older has been suggested to be associated with better gross motor skills [6]. Birth order within a family has also been suggested as a factor that may affect motor competence, with younger siblings of older sisters/brothers displaying better motor performance, compared to only children [26,28]. However, at a US national level, there is a dearth of available data to evaluate gross motor competency and characterize socioeconomic and familial factors associated with gross motor development among young children.

In 2012, the National Health and Examination Survey (NHANES) National Youth Fitness Survey (NNYFS) was jointly implemented with NHANES. In the NNYFS, a representative sample of US children aged 3–5 years participated in a gross motor skill assessment. The assessment provided the first nationally representative data on locomotor and object control skills among young children. Previously, Kit et al. [29] utilized the NNYFS data to describe gross motor skills among US children aged 3 to 5 years. This study reported that gross motor development did not differ by demographic characteristics or weight status, using gross motor score data as continuous variables. However, the study did not examine the characteristics of children “at risk of below average” for gross motor skills [30]. Using the NNYFS data, the first aim of this study was to examine the prevalence of below average gross motor skills in a representative sample of US children aged 3 to 5 years. The second aim was to identify socioeconomic and familial characteristics that are associated with below average at gross motor skills.

## 2. Materials and Methods

### 2.1. Participants

Secondary analysis was conducted using the datasets from the 2012 NNYFS. The NNYFS assessed gross motor skills among children aged 3–5 years, using the Test of Gross Motor Development-Second Edition (TGMD-2). Using a complex staged sampling method and stratifying based on age and sex, a representative sample of 368 children aged 3–5 years at the time of screening was selected for the NNYFS. Of them, 16 participants were ineligible for the examinations because they had physical limitations requiring a wheelchair, amputation of the leg, foot, arm, or hand, paralysis of one or both arms or hands, hand/arm/shoulder/leg surgery in the last 3 months, or a mental impairment. Of the 352 eligible participants, six did not participate in the TGMD-2, and 17 participated in but did not complete the TGMD-2. Excluding those 23 children without complete TGMD-2 data, this report included 329 participants (49.5% girls) aged 3 to 6 years at the time of the TGMD-2 (*n* = 104 at age 3 years, *n* = 115 at age 4 years, *n* = 124 at age 5 years, and *n* = 10 at age 6 years). Compared to the 329 included participants, the 39 excluded participants tended to be younger (*p* = 0.06), but otherwise the distributions of sex, race/ethnicity, and family income were similar.

### 2.2. Measurements

All measurements were conducted in the NNYFS mobile examination center (MEC) across all US states in urban and rural areas. The MEC environment (a single trailer) was designed to accommodate physical activity and fitness examinations and interviews. All participants were asked to dress in comfortable, loose-fitting, short-sleeved or sleeveless shirts or shorts. Shorts and shoes were available for children who did not come appropriately dressed for the examination.

The main outcome of this study was gross motor skills. Gross motor skills were evaluated using the TGMD-2. The TGMD-2 is a norm-referenced measure of common gross motor skills that develop early in life [31]. The TGMD-2 has been validated [1] and widely used [6,9] for gross motor skill assessment. The TGMD-2 is composed of two gross motor subtests: locomotor and object control tests. The locomotor test measures the following six skills: run (4 criteria), gallop (4 criteria), hop (5 criteria), leap (3 criteria), horizontal jump (4 criteria), and slide (4 criteria). The object control test measures the following six skills: striking a stationary ball (5 criteria), dribble (4 criteria), kick (4 criteria), catch (3 criteria), overhand throw (4 criteria), and underhand roll (4 criteria). In the NNYFS, the total time allotment for the TGMD-2 was 15–20 min. The developer of the TGMD-2 provided consultation on conducting the TGMD-2 component. The detailed procedure manual for the TGMD-2 component is publicly available at the NNYFS website [31]. In accordance with the TGMD-2 manual [1], a locomotor skill standard score (range of 1 to 20), an object control skills standard score (range of 1–20), and a gross motor quotient (range of 55 to 150) were calculated. Based on the descriptive rating system suggested in the TGMD-2 manual (the subtest standard score and the gross motor quotient ≤3 and <70 for “very poor”; 4–5 and 70–79 for “poor”; 6–7 and 80–89 for “below average”; 8–12 and 90–110 for “average”; 13–14 and 111–120 for “above average”; 15–16 and 121–130 for “superior”; and 17–20 and >130 for “very superior”, respectively) [1], each of the locomotor skill standard score and the object control skills standard score was dichotomized into lower (≤7, “below average”) and higher (≥8, “average”) [1]. The gross motor quotient was also dichotomized into lower (≤89, “below average”) and higher (≥90, “average”) [1]. “≤Below average” corresponds to <25th percentile.

Socioeconomic and familial characteristics of interest included race/ethnicity, family income, family size, living with other child(ren) in household, acculturation, physical activity behavior, and television (TV)/video viewing. Questions about those characteristics were asked to an adult family member, as a proxy for the participants, during in-person interviews using the Computer-Assisted Personal Interviewing system. For this study, race/ethnicity was categorized into Hispanic, non-Hispanic Black, non-Hispanic White, and other. Family income was categorized into <$25,000, $25,000 to <$75,000, and ≥$75,000. Ratio of family income to poverty (family size-adjusted household income level) was calculated as family income divided by poverty threshold. The poverty threshold accounts for the size of the family and the number of related children under age 18 years. The ratio of family income to poverty was categorized into 0 to <1.0 (low), 1.0 to <3.0 (middle), and ≥3.0 (high). For a family with four household members, including two children under age 18 years, the poverty threshold in 2012 was $23,283. Therefore, a cut-point of 3 for the ratio of family income to poverty was equal to $69,849 for the family with four household members. Family size was dichotomized into ≤4 family members and ≥5 family members. Variables for whether living with other child(ren) ≤5 years old in the same household (yes or no) and whether living with child(ren) aged 6–17 years old in the same household (yes or no) were also created. To examine acculturation, primary language(s) used at home were asked. The primary language(s) used were dichotomized into English only and non-English. To examine participation in daily physical activity, how many days the participant was physically active for a total of at least 60 min per day during the past 7 days was asked. The number of days was dichotomized into ≤6 days and 7 days. Specific types of physical activity engaged in during past 7 days were also asked. Priori analyses (a series of chi-square tests between each of the 32 physical activity types and gross motor skills) revealed that bike riding, scooter riding, soccer, swimming, and trampoline were significantly associated with a higher gross motor quotient, which is supported by a prior publication [27]. Therefore, we created a new variable to indicate whether a participant engaged in bike riding, scooter riding, soccer, swimming, or trampoline in the past week (yes or no). To examine time spent in television (TV)/video viewing, how many hours per day a participant sat and watched TV or videos over the past 30 days was asked. The number of hours spent in watching TV/videos was dichotomized into ≤1 h and ≥2 h, in accordance to the screen time guidelines for children ages 2–5 years (≤1 h) by the American Academy of Pediatrics [32].

In addition, we examined birthweight and weight status. Birthweight was asked during the interview. Birthweight was categorized into <2.5 kg, 2.5 to <4.0 kg, and ≥4.0 kg. Standing height and weight were measured by a trained health technician using a portable stadiometer and a portable digital weight scale. Body mass index (BMI) and sex- and age-specific BMI percentile were calculated based on the 2000 Center for Disease Control and Prevention (CDC) growth charts. Weight status was categorized into healthy weight (<85th BMI percentile), overweight (85th to <95th BMI percentile), and obese (≥95th BMI percentile).

### 2.3. Statistical Analysis

All analyses were conducted incorporating the complex sample design, such as weighting and clustering, using SAS 9.4 (Cary, NC, USA). Descriptive analyses, including mean and 95% confidence interval (CI), were conducted for the gross motor skill outcomes by sex and age. Chi-square tests were conducted between the dichotomized gross motor quotient variable and the characteristics of interest. Two sample *t*-tests were also conducted to compare mean gross motor quotients by the characteristics of interest. A sex- and age-adjusted multivariable logistic regression analysis was conducted to predict a higher gross motor quotient, including predictors that were found to be significant in the chi-square tests. A significance level was set at 0.05 (two-tailed). These analyses were repeated for the locomotor and object control skill outcomes.

## 3. Results

Table 1 presents mean gross motor scores by age and sex. The average gross motor quotient was 95.6 in this representative sample of US children aged 3–5 years. Both locomotor and object control raw scores showed an increasing trend with age. However, their standard scores were similar across age. The locomotor skills standard score was higher among girls aged 4 and 5 years, compared to their boy counterparts.

As shown in Table 2, 33.9% of the sample had a gross motor quotient below average. Lower family size-adjusted family income and living with child(ren) ≤5 years old were associated with a higher gross motor quotient (*p* < 0.05). Those with less TV/video viewing time tended to have a higher gross motor quotient, but it was statistically insignificant (*p* = 0.12). Hispanic children tended to have a higher gross motor quotient, but it was statistically insignificant (*p* = 0.14). In contrast, two-sample *t*-tests showed no significant difference in mean gross motor quotients by the characteristics examined (data not shown).

A multivariable logistic regression model (Table 3) showed that those living at or below the poverty threshold were more likely to have a higher gross motor quotient (odds ratio, OR = 2.76; 95% CI = 1.09–7.00), indicating an inverse association between family income and gross motor development.

Examining the gross motor skill subsets, 24.4% of the sample had a locomotor standard score below average (Table 4). Being a girl, having a lower family size-adjusted household income, and living with child(ren) ≤5 years old were associated with a higher locomotor score (*p* < 0.05). Those with high birthweight (≥4 kg) tended to have a lower locomotor score, but it was statistically insignificant (*p* = 0.06). In the sample, 39.9% had an object control standard score below average. Living with child(ren) aged 6–17 years old was associated with a higher object control score (<0.01). In contrast, living with child(ren) aged ≤5 years old tended to be associated with a lower object control score, but it was statistically insignificant (*p* = 0.10).

Multivariable logistic regression models (Table 5) show that girls were more likely to have a higher locomotor score (OR = 2.17; 95% CI = 1.10–4.25). Those living with child(ren) aged ≤5 years old were more likely to have a higher locomotor score (OR = 2.36; 95% CI = 1.01–5.54). Those living with child(ren) aged 6–17 years old were more likely to have a higher object control score (OR = 1.83; 95% CI = 1.24–2.69).

## 4. Discussion

The current study reports on the gross motor skill levels and associated characteristics in a nationally representative sample of US children age 3 to 5 years old. This study estimated that one in three children aged 3 to 5 years in the US have gross motor skills that are below average. Looking specifically at object control skills, two in five children demonstrated below average skills. Among the socioeconomic and familial factors investigated, we found that children living below the poverty threshold were more likely to have better gross motor skills, indicating an inverse association between family income and gross motor skill development. Girls had better locomotor skills than boys. Living with sibling(s) or other child(ren) aged ≤5 years was associated with better locomotor skills, while living with sibling(s) or other child(ren) aged 6–17 years old was associated with better object control skills.

Globally, several studies [6,9,13,33] have reported on gross motor skill levels among young children using the TGMD-2. Bardid et al. [9] examined gross motor skills in a representative sample of 938 children aged 3 to 5 years in the Flemish region of Belgium in 2012, and showed similar levels of locomotor and object control raw scores, compared to the US-based sample of this current study. Barnett et al. [33] examined gross motor skills among 127 Australian firstborn children aged 5 years sampled from the Melbourne area in 2013–2015, and showed lower locomotor and object control raw scores, compared to the US-based sample of the current study (locomotor raw score of 26.0 in Australia vs. 33.3 in US; object control raw score of 23.3 in Australia vs. 26.2 in US). This difference could be explained in part by the positive sibling effect and the negative sociodemographic status effect that the current study demonstrated. Jiang et al. [13] examined gross motor skills among 60 Chinese children aged 3 to 5 years sampled from the city of Beijing, and reported higher levels of locomotor and object control raw scores in all of the three age groups, compared to the US-based sample of the current study. Because the Chinese study did not report socioeconomic and familial characteristics that could explain the difference, the reason for the difference is largely unknown. However, because the Chinese study categorized children in a 3/4 year old class as 3 years old, children in a 4/5 year old class as 4 years old, and children in a 5/6 year old class as 5 years old, the inclusion of older children in each age group could have resulted in higher gross motor skill raw scores. Tomaz et al. [14] examined gross motor skills among 259 South African children aged 3 to 6 years sampled from both urban and rural areas in 2012 and 2014, and reported higher gross motor skills, compared to the current study (a gross motor quotient below average: 7.0% in South Africa vs. 33.9% in US). Overall, the differences across the studies mentioned above could also potentially represent a population (country)-level difference in gross motor development. Future research should follow to better explain the differences in early childhood gross motor development across populations. Given that, although not compulsory, a majority of US preschool-aged children attend full-day or part-day educational programs (40% for 3-year-olds, 68% for 4-year-olds, 85% for 5-year-olds) [34], the inclusion of fundamental motor skills, such as striking a stationary ball, kick, and catch, in the preschool physical education curriculum could help to overcome a low level of attained gross motor development [35,36].

In terms of a sex difference in gross motor competence, the current study found higher locomotor skills among girls, but no sex difference in object control skills. A review by Barnett et al. [7] summarized the literature suggesting strong evidence that boys have better object control skills, but no evidence for a sex difference in locomotor skills. Both the UK study [11] (369 children aged 4–7 years) and the Belgium study [9] also showed higher object control skills among boys, but no sex difference in locomotor skills. However, Hardy et al. [10] reported higher object skills among boys and higher locomotor skills among girls in a sample of 425 children aged 2 to 6 who attended preschools in Sydney, Australia in 2008. Niemistö et al. [12] reported higher object control skills and higher locomotor skills among girls in a sample of 472 Finnish children aged 5 to 7 years. The Chinese study [13] and the South African study [14] mentioned above showed no sex difference in either locomotor or object control skills. Thus, it appears that the sex difference results remain inconsistent across the studies.

It has commonly been hypothesized that higher socioeconomic status positively impacts gross motor development. Some empirical data have also suggested support for this hypothesis [11,14,25]. For example, Morley et al. [11] reported higher gross motor competence among those with higher socioeconomic status (indicated as a deprivation index) in UK children aged 4–7 years. Similarly, McPhillips et al. [25] reported higher motor competence among Irish students aged 4–5 years in socially advantaged schools, compared to their counterparts in disadvantaged schools. The South African study [14] mentioned above reported that the proportion of those with a gross motor quotient below average was 2.2% for preschoolers in urban high-income areas vs. 6.6% for preschoolers in urban low-income areas. However, the current study revealed a somewhat surprising finding of an inverse association between family income and gross motor skill development, particularly for locomotor skills. Upon further examination of the proportion of children with gross motor quotients ≤79 (“poor gross motor skills”) [1], the trend remained, although the finding was statistically insignificant (poor gross motor skills = 9.4%, 11.3%, and 18.4% for the ratio of family income to poverty <1.0, 1.0 to <3.0, and ≥3.0, respectively; *p* = 0.34). These findings contradict the conventional wisdom that lower socioeconomic status will necessarily lead to disadvantages in gross motor competence. The inverse association has been reported in older literature from the 1960s to the 1970s [37,38] but less so in more recent literature. The inverse association could be in line with the prior observation that higher parental social status was associated with later attainment of milestones related to toilet training [39], although the mechanism is not yet well understood. It is plausible that in some cultures or societies, increasing affluence is associated with missed opportunities for gross motor activities, which, in turn, could lead to later attainment of gross motor skills. It should also be noted that social class or socioeconomic status were operationalized in different ways across studies (e.g., father’s occupation, maternal education, housing, family income, deprivation index). As such, more research is needed to help clarify the influence of socioeconomic status on motor competence in early childhood.

The current study demonstrated that living with young sibling(s) is associated with better locomotor skills and living with older sibling(s) aged 6–17 years is associated with better object control skills. These finding are in line with the literature suggesting that siblings play an important role in early childhood development [40]. The sibling effect could exist partly because parents with several children in the home may be more occupied, and thus they inadvertently call upon their preschool-aged children to be more independent in all areas of development, including motor competencies. In contrast, parents of an only child may tend to be overprotective toward their child, thereby affording fewer opportunities for their child to effectively practice their motor skills. All of these are hypotheses that would require further testing to confirm.

Interestingly, this study suggests that the age of siblings influences different types of gross motor skills: better locomotor skills with young siblings and better object control skills with elder siblings. For preschool-aged children, simply being with another young child has been shown to facilitate more locomotor-related physical activity, such as running, compared to being alone or being with older children [41]. Thus, living in a home with other young children could facilitate improved locomotor development. When in the presence of an older sibling, young children often copy his/her motor behaviors and are more likely to explore the surrounding environment and objects [42]. The presence of older siblings may provide a basis for learning by example, thereby prompting and encouraging motor development through role modeling [42,43]. Studies have shown increased imitation among young children with older siblings, though this has been seen more so for play than in areas specific to motor development [44]. Furthermore, older siblings frequently engage in teaching behaviors with younger siblings, engaging in a relationship that becomes more bidirectional and cooperative with time [45], and some studies have indicated that with descending birth order, cooperative abilities increase [46]. It could be hypothesized that older siblings above the age of 6 years are more proficient in teaching their preschool-aged siblings new ball skills, for example, and preschool-aged children may be socially motivated to master these skills in order to encourage continued back-and-forth play exchanges with their older siblings.

This study is significant in that it reports the most recent gross motor skill data and associated factors in a representative sample of US children aged 3–5 years. However, several limitations should be acknowledged. First, this secondary analysis did not include potentially important confounding factors, such as exposures outside of the home (involvement in preschool, daycare, community recreational activities, etc.), premature birth, and genetic predisposition to gross motor competencies [47]. Second, there may be other elements of the family structure beyond household size and presence of siblings (e.g., single-parent family) that are important factors influencing gross motor development. However, we could not examine their effects, because such data were unavailable. Third, despite the use of the most recent gross motor data available from a nationally representative sample, caution is needed to interpret the results, given that this study used data collected eight years ago in 2012 and as such, child development and family environments may have changed during this period.

## 5. Conclusions

In conclusion, this study estimated that one in three US children age 3 to 5 years old had a below average gross motor quotient. In the two gross motor subsets, one in four US children had below average locomotor standard scores and two in five had below average object control standard scores. Girls and those with lower family income presented higher locomotor skills. Living with siblings also appears to facilitate the acquisition of gross motor skills.

## Figures and Tables

**Table 1:** Scores of gross motor development.

Age	Sex (*n*)	Locomotor Raw Score	Object Control Raw Score	Locomotor Standard Score Weighted Mean (95% CI)	Object Control Standard Score	Gross Motor Quotient
3–5 years	Both (329)	28.3 (27.2–29.4)	21.1 (20.4–21.8)	10.0 (9.6–10.4)	8.5 (8.2–8.8)	95.6 (93.9–97.3)
3 years	Both (88)	19.9 (17.6–22.1)	15.2 (13.8–16.6)	9.3 (8.6–10.1)	8.6 (8.1–9.2)	93.8 (90.6–97.0)
	Boy (43)	19.4 (16.7–22.2)	16.9 (14.7–19.1)	9.2 (8.3–10.1)	8.7 (7.8–9.5)	93.7 (89.0–98.4)
	Girl (45)	20.3 (17.8–22.9)	13.4 (12.3–14.6)	9.5 (8.6–10.3)	8.5 (8.1–9.0)	94.0 (90.6–97.04)
4 years	Both (111)	29.5 (27.5–31.4)	20.2 (19.0–21.3)	10.6 (9.9–11.2)	8.5 (8.1–8.9)	97.2 (94.9–99.4)
	Boy (57)	27.7 (24.3–31.1)	21.5 (18.9–24.0)	10.0 (8.9–11.2)	8.4 (7.6–9.3)	95.4 (90.2–100.5)
	Girl (54)	31.0 (28.8–33.2)	19.0 (16.8–21.2)	11.1 (10.3–11.8)	8.5 (7.7–9.3)	98.8 (95.3–102.3)
5 years	Both (130)	33.3 (31.6–35.0)	26.2 (24.2–28.3)	10.0 (9.4–100.6)	8.5 (7.9–9.2)	95.4 (92.1–98.7)
	Boy (66)	31.6 (29.8–33.4)	28.2 (25.7–30.7)	9.3 (8.7–9.8)	8.6 (7.8–9.3)	93.5 (90.0–97.1)
	Girl (64)	35.3 (32.4–38.2)	24.0 (21.6–26.5)	10.7 (9.7–11.8)	8.4 (7.5–9.4)	97.6 (92.0–103.1)

CI, confidence interval.

**Table 2:** Bivariate analyses between participant characteristics and gross motor skill level.

		Lower Gross Motor Quotient	Higher Gross Motor Quotient	*p*-Value
		Unweighted *n* (Weighted %)	Unweighted *n* (Weighted %)	
Total		112 (33.9)	217 (66.1)	
Sex	Boy	61 (37.5)	105 (62.5)	0.17
	Girl	51 (30.3)	112 (69.7)	
Age	3 years	35 (43.1)	53 (56.9)	0.20
	4 years	33 (28.9)	78 (71.1)	
	5 years	44 (32.2)	86 (67.8)	
Race/ethnicity	Hispanic	29 (24.3)	81 (75.7)	0.14
	Non-Hispanic Black	25 (35.6)	45 (64.3)	
	Non-Hispanic White	48 (35.8)	83 (64.2)	
	Other	10 (52.0)	8 (48.0)	
Family income	$0 to $24,999	26 (22.9)	86 (77.1)	0.06
	$25,000 to $74,999	45 (38.2)	69 (61.8)	
	≥$75,000	32 (39.5)	51 (60.5)	
Ratio of family income to poverty *	<1.0 (low)	26 (21.8)	93 (78.2)	<0.01
	1 to <3.0 (middle)	41 (37.0)	62 (63.0)	
	≥3.0 (high)	36 (42.5)	51 (57.5)	
Household size	≤4 people	58 (37.6)	111 (62.4)	0.10
	≥5 people	44 (29.3)	106 (70.7)	
Living with Child(ren) ≤ 5 years old	No	68 (39.9)	111 (60.1)	0.04
	Yes	44 (27.5)	106 (72.5)	
Living with Child(ren) 6–17 years old	No	52 (35.7)	91 (64.3)	0.46
	Yes	60 (32.4)	126 (67.6)	
Language spoken at home	English only	77 (34.0)	147 (66.0)	0.98
	Non-English	36 (33.8)	70 (66.2)	
Daily ≥60 min of PA	No	18 (30.2)	41 (69.4)	0.63
	Yes	94 (34.7)	176 (65.3)	
Engaging in gross motor-related PA **	No	67 (41.5)	99 (58.5)	0.02
	Yes	45 (27.2)	118 (72.8)	
TV or video viewing	≤1 h/day	49 (29.8)	107 (70.2)	0.12
	≥2 h/day	63 (37.9)	110 (62.0)	
Birthweight	<2.5 kg	6 (26.1)	17 (73.9)	0.78
	2.5 to <4 kg	95 (34.0)	175 (66.0)	
	≥4 kg	11 (37.9)	25 (62.1)	
Weight status	Healthy weight	79 (33.5)	147 (66.5)	0.89
	Overweight	14 (33.5)	36 (66.5)	
	Obese	19 (36.5)	34 (63.5)	

* missing *n* = 20. PA, physical activity. ** gross motor-related PA included bike riding, scooter riding, soccer, swimming, or trampoline.

**Table 3:** Odds ratios of higher gross motor skills.

Predictors	OR	Lower Limit of 95% CI	Upper Limit of 95% CI
Sex: girl vs. boy	1.40	0.82	2.40
Age: 4 vs. 3 years	1.86	0.97	3.58
Age: 5 vs. 3 years	1.44	0.77	2.69
Ratio of family income to poverty: 1.0 to <3.0 vs. ≥3.0	1.36	0.54	3.47
Ratio of family income to poverty: <1.0 vs. ≥3.0	2.76	1.09	7.00
Living with child(ren) ≤5 years old: yes vs. no	1.57	0.81	3.06
Engaging in gross motor-related PA: yes vs. no	1.93	1.04	3.58

CI, confidence interval; PA, physical activity; OR, odds ratio.

**Table 4:** Bivariate analyses between participant characteristics and locomotor and object control scores.

		Locomotor			Object Control		
		Lower Locomotor Scores	Higher Locomotor Scores	*p*-Value	Lower Object Control Scores	Higher Object Control Scores	*p-*Value
		Unweighted *n* (Weighted %)	Unweighted *n* (Weighted %)		Unweighted *n* (Weighted %)	Unweighted *n* (Weighted %)	
Total		80 (24.4)	249 (82.7)		111 (39.9)	218 (70.6)	
Sex	Boy	50 (31.1)	116 (68.9)	0.01	56 (34.4)	110 (65.6)	0.91
	Girl	30 (17.6)	133 (82.4)		55 (34.9)	108 (65.1)	
Age	3 years	24 (29.1)	64 (70.9)	0.66	20 (26.8)	68 (73.2)	0.40
	4 years	28 (22.8)	83 (77.2)		41 (39.1)	70 (60.9)	
	5 years	28 (22.7)	102 (77.3)		50 (35.9)	80 (64.1)	
Race/ethnicity	Hispanic	20 (16.9)	90 (83.1)	0.15	33 (28.3)	77 (71.7)	0.48
	Non-Hispanic Black	14 (18.8)	56 (81.2)		23 (34.4)	47 (65.6)	
	Non-Hispanic White	37 (27.2)	94 (72.8)		48 (36.9)	83 (63.1)	
	Other	9 (44.8)	9 (55.2)		7 (40.5)	11 (59.5)	
Family income	$0 to $24,999	20 (17.4)	92 (82.6)	0.12	31 (29.4)	81 (70.6)	0.49
	$25,000 to $74,999	27 (23.6)	87 (76.4)		45 (38.6)	69 (61.4)	
	≥$75,000	27 (32.3)	56 (67.7)		27 (35.6)	56 (64.4)	
Ratio of family income to poverty *	<1.0 (low)	21 (17.3)	86 (82.7)	0.03	31 (27.9)	88 (72.1)	0.19
	1 to <3.0 (middle)	23 (21.3)	80 (78.7)		40 (40.5)	60 (59.5)	
	≥3.0 (high)	30 (34.7)	57 (65.3)		29 (35.6)	58 (64.4)	
Household size	≤4 people	46 (26.7)	133 (73.3)	0.29	67 (37.6)	112 (62.4)	0.23
	≥5 people	34 (21.6)	116 (78.4)		44 (30.8)	106 (69.2)	
Living with Child(ren) ≤ 5 years old	No	53 (32.5)	126 (67.5)	<0.01	54 (30.9)	125 (69.1)	0.10
	Yes	27 (15.7)	123 (84.2)		57 (38.6)	93 (61.4)	
Living with Child(ren) 6-17 years old	No	34 (22.6)	109 (77.4)	0.38	57 (41.3)	86 (58.7)	<0.01
	Yes	46 (26.0)	140 (74.0)		54 (28.9)	132 (71.1)	
Language spoken at home	English only	54 (24.3)	170 (75.7)	0.92	75 (34.6)	149 (65.4)	0.96
	Non-English	26 (24.8)	79 (75.2)		36 (34.8)	69 (65.2)	
Daily ≥60 min of PA	No	13 (22.4)	46 (77.6)	0.80	20 (35.5)	39 (64.5)	0.91
	Yes	67 (24.8)	203 (75.2)		91 (34.4)	179 (65.6)	
Engaging in gross motor-related PA **	No	67 (41.5)	99 (58.5)	0.02			
	Yes	48 (30.3)	118 (69.7)	0.04	60 (38.4)	106 (61.6)	0.26
TV or video viewing	≤1 h/day	37 (23.0)	119 (77.0)	0.51	55 (33.1)	118 (66.9)	0.51
	≥2 h/day	43 (25.9)	130 (74.1)		56 (36.1)	100 (63.9)	
Birthweight	<2.5 kg	5 (23.6)	18 (76.4)	0.06	8 (42.3)	15 (57.7)	0.37
	2.5 to <4 kg	68 (24.1)	202 (75.9)		89 (32.5)	181 (67.5)	
	≥4 kg	7 (27.1)	29 (72.9)		14 (44.7)	22 (55.3)	
Weight status	Healthy weight	59 (25.2)	167 (74.8)	0.87	78 (35.0)	148 (65.0)	0.97
	Overweight	9 (21.4)	41 (78.6)		17 (34.7)	33 (65.3)	
	Obese	12 (24.2)	41 (75.8)		16 (32.9)	37 (67.1)	

* missing *n* = 20. PA, physical activity. ** gross motor-related PA included bike riding, scooter riding, soccer, swimming, or trampoline.

**Table 5:** Odds ratios of a higher locomotor score and a higher object control score.

Predictors	Locomotor			Object Control		
	OR	Lower Limit of 95% CI	Upper Limit of 95% CI	OR	Lower Limit of 95% CI	Upper Limit of 95% CI
Sex: girl vs. boy	2.17	1.10	4.25	1.01	0.67	1.53
Age: 4 vs. 3 years	1.45	0.57	3.72	0.53	0.23	1.26
Age: 5 vs. 3 years	1.23	0.44	3.41	0.60	0.18	1.98
Ratio of family income to poverty: 1.0 to <3.0 vs. ≥3.0	2.07	0.87	4.91	NA	NA	NA
Ratio of family income to poverty: <1.0 vs. ≥3.0	2.51	0.94	6.70	NA	NA	NA
Living with child(ren) ≤5 years old: yes vs. no	2.36	1.01	5.54	NA	NA	NA
Living with child(ren) 6–17 years old: yes vs. no	NA	NA	NA	1.83	1.24	2.69
Engaging in gross motor-related PA: yes vs. no	1.83	0.94	3.57	NA	NA	NA

CI, confidence interval; NA, not applicable because the parameter was not included in the multivariable logistic regression model; PA, physical activity; OR, odds ratio.
